# New Directions for Natural Killer T Cells in the Immunotherapy of Cancer

**DOI:** 10.3389/fimmu.2017.01480

**Published:** 2017-11-08

**Authors:** Luc Teyton

**Affiliations:** ^1^Department of Immunology and Microbiology, Scripps Research Institute, La Jolla, CA, United States

**Keywords:** natural killer T cells, endogenous ligands, single cell analysis, anti-PD1 treatment, vaccines

## Abstract

Natural killer T (NKT) cells have been placed at the interface between innate and adaptive immunity by a long series of experiments that convincingly showed that beyond cytokine secretion and NK cell recruitment, NKT cells were coordinating dendritic cell and B cell maturation through direct membrane contacts and initiate productive responses. As such, NKT cells are the cellular adjuvant of many immune reactions and have functions that go much beyond what their name encapsulates. In addition, the initial discovery of the ligands of NKT cells is deeply linked to cancer biology and therapy. However, for a host of reasons, animal models in which agonists of NKT cells were used did not translate well to human cancers. A systematic reassessment of NKT cells role in tumorigenesis, especially spontaneous one, is now accessible using single cell analysis technologies both in mouse and man, and should be taken advantage of. Similarly, the migration, localization, phenotype of NKT cells following induced expansion after injection of an agonist can be examined at the single cell level. This technological revolution will help evaluate where and how NKT cells can be used in cancer.

## Introduction

Natural killer T (NKT) cells were discovered more than 30 years ago as a small population of double negative or CD4+ T cells in the mouse and characterized by the usage of a unique T cell receptor (TCR) α chain with an invariant CDR3 segment. The same population was subsequently isolated from human blood and shown to express the same TCRα chain as the mouse, instantly raising the attention of researchers around the world as conservation of T cell population across distant species usually correlates to essential functions (1). In addition, it was also reasonable to assume that a semi-invariant TCR would recognize a limited set of antigens. The discovery of the first of these antigens took 10 years and serendipity, and linked forever NKT cells with cancer biology (2). Indeed, α-galactosylceramide (αGalcer) was isolated by a team of researchers at Kirin Pharmaceuticals who were seeking antitumor properties in natural compounds. The extracts from Agelas Mauritanus proved great antitumor effects in a melanoma model that they used for screening. The chemical nature of the natural product responsible for the activity was surprising and related to species that were isolated in 1989 by Mangoni and collaborators, from *Amphimedon viridis*, a marine sponge, and were identified as α-glucosamine ceramides (3). The connection of this new glycolipid, αGalcer, with NKT cell was also very circumstantial as in the same year, the crystal structure of CD1d was elucidated and revealed the lipid binding properties of this class of MHC-like molecules (4). With the rapid development of CD1d- αGalcer tetramers, a field was born as NKT cells were now accessible in and *ex vivo* for studies. The discovery that CD1d was presenting other lipids and glycolipids to non-semi-invariant T cells was in the shadow of the semi-invariant NKT cells for years and led to a rather incongruous and inappropriate split in terminology of lipid-specific T cells with type 1 and type 2 NKT cells for the semi-invariant and non-semi-invariant cells, respectively. The NKT name is related to the expression of NK markers by NKT cells but this name brings numerous confusions between the two cell types, while type 2 NKT cells appear to be mainstream T cells (5). In any case, we will only discuss the classic type 1 NKT cells in this communication.

**Figure 1:**
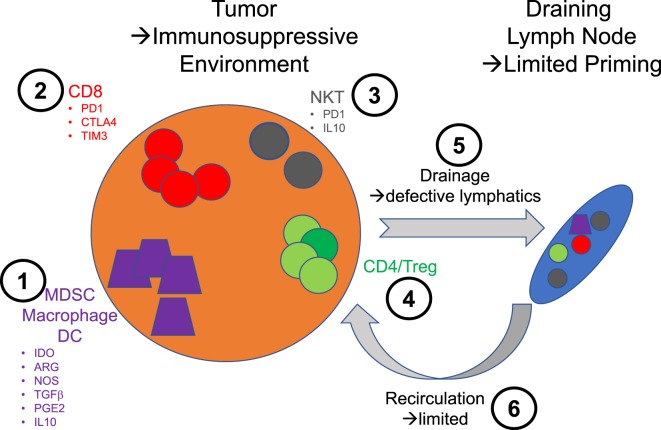
Schematic illustration of the various therapeutic intervention nodes that are relevant to the use of natural killer T (NKT) cells in immunotherapy. **1**: Monocyte lineage—these cells are essential to sample tumors and traffic to the lymph node to prime immune responses. The secretion of indoleamine 2,3-dioxygenase by the tumor cells promotes the emergence of a suppressive phenotype linked to the downstream expression of immunosuppressive mediators such as arginase (ARG), nitric oxide synthase (NOS), transforming growth factor β (TGFβ), prostaglandin E2 (PGE2), and IL10. **2**: CD8 T cells—the exhausted phenotype of these cells in tumor is directly exploited for intervention with checkpoint inhibitors. **3**: NKT cells—in very much the same way CD8 T cell responses are improved by checkpoint blockade, NKT cells could benefit from the same treatment. In addition, the blockade of some of the immunosuppressive factors they may produce could be beneficial. **4**: CD4 T cells—there is not to this day therapies intervening directly on this population of T cells to enhance their effector function or block their regulatory activities. **5**: Lymphatics—the lymphatic architecture of tumors is notably compromised and likely contributes to poor priming against tumors. **6**: Blood vessels—the normal circulation is similarly aberrant and most likely compromises the access of effector cells to tumors.

As mentioned, the discovery of αGalcer in the context of a cancer program focused the attention of many on the potential linkage of NKT cells with the biology of cancer. The demonstration of a potent antimelanoma activity in mice and the analysis of some of the mechanisms that led to this response such as the ability of some NKT cells to lyse target cells, enticed clinicians to trying αGalcer as monotherapy in advanced cancers (6). In these trials, if the bioactivity of the compound could be demonstrated based on the expansion of the blood NKT cell population, no clinical benefit could be shown (7–9). As often in the field of immunotherapy, initial failures greatly diminish the appetite of companies and clinicians to use a particular compound, and the consideration of αGalcer for cancer treatment has greatly diminished if not vanished. However, the biology of NKT cells, the identification of their natural ligands, the behavior of their agonists *in vivo*, and the entire field of immunotherapy have made huge progress over the past decade and new approaches that would put NKT cells at the center of the treatment of cancer are emerging (Figure 1). This is precisely what we will discuss and place in the context of understanding the particular role of NKT cells in tumorigenesis in both mouse and man.

## Definition of the NKT Cell Populations in Mouse and Man, Health and Diseases

As a preamble, it is important to mention that a substantial part of the NKT cell-cancer field has been shrouded in its early years by discrepancies and controversies that have to do with the relationship between cancer and the presence of NKT cells. In mouse models where NKT cells can be removed by either deletion of CD1d, the antigen-presenting molecule, or Jα18, the junctional segment of NKT cells, some have found an increase in MCA-induced tumors in the absence of NKT cells (10), whereas other have not (11). However, the dramatic effect of NKT cell activation on the fate of metastatic melanoma in mice kept the interest high enough for clinical trials to be carried out with the outcome that we have just mentioned. This discrepancy between mouse studies and clinical trials could be used for the sake of making the argument that mouse models are poorly predictive or human studies, but is this true? The mouse models offer the advantage of speed and the luxury of accessing every organ and lymph node during the course of a disease that we initiate. We often overlook the fact that the B16 melanoma model in its subcutaneous solid tumor or metastatic versions are acute cancers, a situation exceptionally seen in human. So, in this case, it is not the mouse biology that trumps us but the model we decide to use for practical and financial reasons. Spontaneous tumors would and should offer a much better model to evaluate NKT cells in the control of tumorigenesis and the NKT cell-based therapies but they take a long time. However, even in the case of the B16 melanoma model, most of the studies are shortcoming in understanding the role of NKT cells. We live on a model in which the secretion of IFNγ by NKT cells explains all the therapeutic effects of αGalcer by allowing not only the maturation of dendritic cells but a large recruitment of NK cells and cytotoxic activities (12). However, little is known about where NKT cells are localized and what their function is before αGalcer is injected. Why cannot they control tumor seeding and development? Are NKT cells exclusively in the lymph node or also in the tumor? What gene expression profiles have those NKT cells? Are they tissue- or blood-resident? Are some of them negative regulators of immune responses? Why do not they initiate a spontaneous immune response and tumor rejection? In all cases, the difficulty to study these functions and mechanisms is the paucity of the NKT cell population even in mice. In human, it is often brought up that NKT cell numbers vary greatly in the general population, spanning at least three orders of magnitude. However, this observation pertains only to peripheral blood NKT cells and variability in other tissue-resident NKT cells has never been systematically documented. The issue that is often overlooked is that NKT cells are tissue-resident and organ-specific (13), and that circulating cells are simply resident of the circulatory system, not merely circulating to the next organ. The molecular basis of this tissue residency is mostly unknown at the exception of the relationship between CXCR6 expression and liver NKT cells (14). What makes an NKT cell resident of a lymph node at a particular location is a field to be explored. In any instance, these considerations are key to understanding what NKT cells do in the vicinity of a tumor, e.g., the lymph node, or within the tumor environment when they are found locally.

## Single Cell Studies

An exhaustive description of NKT cell subsets has been done over the past decade: NKT1, NKT2, NKT17, NKTreg, double negative, CD4+ (1). This diversity demonstrates the plasticity of T cells and the ability of specifying function in various environments. In relationship to cancer, no clear phenotyping has emerged for NKT cells that are inside the tumor and in draining lymph nodes but unexplained observations have been noted in relationship to phenotype; for instance, it appeared that only DN NKT cells from the liver could protect against MCA tumor development, while spleen and thymic NKT cells could not (15). Therefore, it seems obvious that a detailed description of the various NKT cells in the local environment of tumors must be carried out to understand the fundamental role of NKT cells and tumorigenesis. Because NKT cells are so few, classical approaches cannot be used to address this question. However, we have stepped into the era of single cell analysis and pathway mapping, and the topic “NKT cells and cancer” is ideally suited for applying targeted single cell (SC) gene expression profiling by Q-PCR and systemic gene expression profiling by SC RNA-seq techniques. Microfluidics have made these techniques amenable to both mouse and human studies (16, 17), and in the case of NKT cells, the exceptional quality of the CD1d tetramers make the step of single cell isolation relatively trivial. In addition to pure phenotypic assignment to one of the subsets that we have just cited, RNAseq allows to map pathways that are up- or downregulated and gives a deep understanding of the biology that the isolated cells undergo at the time of sampling. It might not be surprising if these studies uncover an anergic state or a suppressive phenotype for intra-tumoral and draining lymph nodes NKT cells. For instance, the increased expression of PD1 could explain anergy and would beneficiate from anti-PD1 interventions (see below). Such studies and the head to head comparison between mouse and man would allow the best possible translational research in the field. In addition, the same techniques could be used to follow therapeutic administration of NKT cell agonists and examine circulating as well as NKT cells recovered from biopsies.

## Overcoming Anergy and Activation Induced Cell Death

The systemic delivery of agonists of NKT cells has been shown to lead to a depletion in numbers and function of NKT cells, both in the mouse and man (18, 19). One obvious reason for dwindling numbers post-αGalcer administration is activation induced cell death by overstimulation, αGalcer being a very potent ligand *in vitro* and *in vivo*. In human, in the course of a phase 1 clinical trial for hepatitis B, our group discovered that maximal effects were obtained at 2 μg IM and followed by a long-term depletion of peripheral NKT cells (18). Concurrently, it has been shown that higher dosage was accompanied by systemic adverse effects related to liver delivery and IFNγ release. It is obvious that the stunning of the NKT cell compartment by a single injection would be detrimental to therapeutic schemes in which multiple rounds of administration of the therapy, e.g., NKT cell agonist or NKT cell agonist + antigen, would be beneficial. As such, it is highly unlikely that there is a future for therapies that would use αGalcer or similar agonists by itself or simply mixed with an antigen in the formulation of a therapeutic vaccine.

A second issue that might be pertinent to the relationship of NKT cells with cancer, is that there is a possibility that tumorigenesis is accompanied by a chronic low level stimulation of NKT cells and their exhaustion (20). A similar phenomenon has been noted in chronic infections such as HIV and hepatitis B (21), and correlated to an increase PD1 expression. Surprisingly, the NKT cell PD1 expression levels have not been examined yet in cancer patients. The broad use of anti-PD1 therapies creates the perfect opportunity to examine the status of NKT cells before and during the course of treatment. Would PD-1 be increased on NKT cells during malignancies, it might indicate that the manipulation of NKT cells during anti-PD1 antibody treatment might contribute to increasing the effects of this type of immunotherapy. Indeed, it would seem logical that NKT cells engagement would improve antigen presentation and T cell recruitment, therefore enhancing the expansion of anti-tumor T cells.

## Clinical Monitoring. Beware of the Blood Numbers

As mentioned above, both in mouse and man, we have used circulating NKT cell numbers as a surrogate for local effects but no study has systematically examined the correlation between peripheral and local expansion of NKT cells. The possibility of discrepancy between compartments was documented in the mouse when we studied glycolipid transport. Indeed, in the absence of FAAH, a αGalcer lipid binding protein, systemic transport of αGalcer is greatly impaired but local effects are increased (22). Given the alterations of vascularization of tumors and their vicinity, it is difficult to assume that NKT cells agonists will be delivered locally with the same pharmacological parameters than in normal tissues. This possible dissociation of effects between organs should be kept in mind when trying to understand low circulating NKT cell numbers and cancer, even though in the case of neck and head malignancies it appears that low numbers and poor prognosis are correlated (23). Answering this concern is not trivial even in animal models as the recovery of NKT cells from various tissues is illustrious for its inconsistency and variability. Once again, SC analysis should be helpful to investigate some of these issues.

## Use Endogenous NKT Cell Ligands

In natural circumstances, NKT cells initiate activation when their TCR encounters endogenous ligands and then sustain activity on cytokine-mediated signaling. The critical TCR engagement step is mediated by a strong agonist, and is brief as one would expect to avoid overstimulation and cell death (24). The stunning or disappearance of NKT cells mediated by the production of endogenous ligands such as after TLR engagement, have never been noted. It appears that endogenous NKT cell activation is mainly controlled by the availability of ligands and that degradation of stimulatory α-linked glysosylceramides is key to this tight regulation (24). Three enzymes are critical to this catabolism, acid ceramidase, ASAH1, responsible for the first step of degradation, acid α-galactosidase, GLA, and acid α-glucosidase, GAA, both responsible for the removal of the head glycan and the production of free sphingosine. It is notable that during TLR-mediated activation of dendritic cells, all three enzymes are briefly down-regulated before returning to normal levels after about 2 h (25). In addition, we have shown that the chemical blockade of these enzymes increased the stimulatory capacity of dendritic cells and more importantly was efficient *in vivo* at expanding NKT cell populations over prolonged periods of time (Luc Teyton, unpublished). It is also of interest to note that ASAH1 inhibitors have been developed in the context of cancer therapy as chemosensitizing agents based on the hypothesis that ceramides were promoting cell death by apoptosis (26). We would argue that some of the effects that have been observed using these inhibitors are directly linked to the effects on NKT cells. This hypothesis will be interesting to examine in patients receiving anti-ASAH1 inhibitors. It will also be of interest of testing the *in vivo* effects of GLA and GAA inhibitors alone and in combination with ASAH1 inhibitors with respect to expanding NKT cell populations without inducing cell death. Some of these inhibitors have been developed as folding-inducers in some of the forms of GLA and GAA deficiencies and appear safe for use in human. This concept of manipulating a cell population by tuning the availability of it endogenous ligand has never applied in any form of immunotherapy so far; the NKT cell system might offer the first opportunity. Finally, it would also be of great interest to appreciate whether some tumors, in addition to expressing CD1d, might express NKT cell ligands and in what amount. Local overproduction could result in the disappearance or functional anergy of local NKT cells.

## Other Potential Avenues of Using NKT Cells in Immunotherapy

Conceptually, and before we really understand the position of NKT cells in cancer and tumorigenesis, a large number of potential therapeutic avenues focused on NKT cells could be explored (Figure 1). The *in vitro* expansion of autologous NKT cells and reinfusion are practically achievable, but one could argue that in the absence of understanding the key mechanisms to NKT cell organ residency, the exercise will be futile, cumbersome and expensive. Similarly, it could be interesting to explore the potential of CAR T cells made with NKT cell receptors or the Fab of an anti-CD1d-αGalcer antibody, to hardwire recognition and killing; however, this approach could only be tested for tumors expressing CD1d. The usage of αGalcer and related compounds in the context of therapeutic vaccines is more mainstream and would simply take advantage of the remarkable adjuvant properties of NKT cells. The administration of autologous dendritic cells loaded with antigen and αGalcer has been shown effective in mouse models (27) and could ideally be tested to potentiate vaccines such as Provenge™ (7). In addition, this mode of delivery of αGalcer bypasses systemic and liver delivery and as such should be devoid of side effects. On the other hand, direct administration of an antigen mixed with αGalcer would expose the patient to this undesirable stimulation of all NKT cells in his/her body; it cannot be conceived without controlling local delivery and limiting or eliminating systemic transport of αGalcer. With this in mind, direct coupling to the antigen or to particles, or unique routes of administration such as intradermal or intranasal, have been explored with some success to control this issue.

## Conclusion

As often in translational research, the urgency of obtaining results combined with the infatuation of each researcher with his/her favorite cell type, leads to disappointing results. Taking a step back is often granted and querying additional basic knowledge never ill-advised. With the arising of SC technologies, we are today in a unique position of understanding what NKT cells do in the course of tumorigenesis and what role they play together with adaptive immunity to reject cancer cells. This knowledge will be essential to manipulate and utilize NKT cells in the arsenal of immunotherapy.

## Author Contributions

The author confirms being the sole contributor of this work and approved it for publication.

## Conflict of Interest Statement

The author declares that the research was conducted in the absence of any commercial or financial relationships that could be construed as a potential conflict of interest.
